# Better adherence to the life's essential 8 can reduce the risk of gallstone disease: mediated by inflammation and oxidative stress

**DOI:** 10.3389/fpubh.2026.1836977

**Published:** 2026-06-04

**Authors:** Xinqiang Chen, Rudan Hong, Ran Liu, Junmin Zhou, Chuanwen Fu, Jia Zeng, Jianqin Zhong, Liling Chen, Xunfu Zhong, Yangji Baima, Zhuohang Che, Ying Qian, Qiong Meng, Jianzhong Yin

**Affiliations:** 1 School of Public Health, Kunming Medical University, Kunming, China; 2 Zigong Center for Disease Control and Prevention, Zigong, China; 3 School of Public Health, Dali University, Dali, Yunnan Province, China; 4 West China School of Public Health and West China Fourth Hospital, Sichuan University, Chengdu, China; 5 Baoshan Center for Disease Control and Prevention, Baoshan, China; 6 School of Public Health, Key Laboratory of Environmental Pollution Monitoring and Disease Control, Ministry of Education, Guizhou Medical University, Guiyang, China; 7 Chongqing Municipal Center for Disease Control and Prevention, Chongqing, China; 8 Wuhou District Center for Disease Control and Prevention, Chengdu, China; 9 School of Medicine, Tibet University, Lasa, China

**Keywords:** life's essential 8, gallstone disease, inflammation, oxidative stress, the China Multi-Ethnic Cohort

## Abstract

**Background:**

This study aims to systematically investigate the association between the life's essential 8 (LE8) and gallstone disease (GSD), and further examine whether inflammation and oxidative stress mediate this relationship, so as to provide more scientific evidence for developing targeted preventive measures against GSD.

**Methods:**

This is a cross-sectional study utilizing baseline survey data from the China Multi-Ethnic Cohort (CMEC). Multivariate logistic regression and multiple linear regression models were employed to investigate the relationships among the LE8 score, inflammatory and oxidative stress biomarkers, and GSD. restricted cubic spline (RCS) regression analysis was employed to further investigate potential nonlinear relationships between LE8 and GSD. Additionally, the “mediation” package in R was utilized to determine whether the association between LE8 and GSD was mediated by inflammation and oxidative stress. Finally, subgroup and interaction analyses were conducted to understand the specific effects of LE8 on GSD across different populations.

**Results:**

This study ultimately included 85,302 subjects with complete data. The overall prevalence of GSD was 7.26%. In a multivariate logistic regression model fully adjusted for age, gender, ethnicity, household registration, marital status, region, education level, and annual household income, the LE8 score showed a significant negative correlation with GSD risk (For each 10-point increase in LE8: OR = 0.833, 95% CI: 0.815–0.852, *P* < 0.001), and this association exhibited a significant nonlinear trend (*P* for nonlinear = 0.005). The results of the mediation analysis further indicated that the relationship between LE8 and GSD was partially mediated by inflammation and oxidative stress, with mediation proportions of 2.85 and 5.21%, respectively. Additionally, subgroup and interaction analyses indicated that the preventive effect of increased LE8 score on GSD was more pronounced in individuals under 50 years old, females, and those of Han ethnicity.

**Conclusion:**

Among the Chinese adult population, the LE8 score exhibited a significant nonlinear inverse dose-response relationship with GSD. This inverse association was partially mediated by improving the body's inflammatory and oxidative stress responses. These new findings contribute to developing novel and more targeted public health interventions for the prevention and management of GSD.

## 1 Introduction

Gallstone disease (GSD) is a condition in which excessively high levels of cholesterol or bilirubin in bile form stones within the gallbladder or biliary tract (1). In recent years, with socioeconomic development and changes in human lifestyles, GSD has gradually shown a trend toward global prevalence (2), particularly in Western developed countries such as the United States and Europe, where it affects nearly one-fifth of the population (3). According to the latest disease burden analysis, GSD causes economic losses of up to $17 billion annually in the United States alone (4). Even more concerning is the growing body of evidence indicating that GSD also increases the risk of cardiovascular disease (CVD) and multiple malignant tumors, including liver cancer, biliary tract cancer, pancreatic cancer, colorectal cancer, and kidney cancer (5–8). These conditions further exacerbate the overall health status and quality of life for GSD patients. Therefore, gaining a thorough understanding of modifiable risk factors associated with GSD holds significant public health implications for developing more targeted intervention strategies for GSD.

Currently, GSD is widely recognized as the result of interactions between genetic and environmentally modifiable factors (9). However, relevant studies indicate that genetic factors account for only 25% of the overall risk of developing GSD (10), and this genetic risk can be partially offset by adopting a healthier diet (11). Therefore, the relationship between various modifiable lifestyle behaviors or physiological conditions and GSD has become a focal point for numerous researchers. In this regard, the life's essential 8 (LE8) serves as a comprehensive metric introduced by the American Heart Association (AHA) in 2022 (12). It holistically assesses or monitors cardiovascular health (CVH) in individuals and populations through eight modifiable behavioral and physiological indicators: diet, physical activity (PA), nicotine exposure, sleep, body mass index (BMI), blood pressure, blood glucose, and blood lipids (12). As a key primary prevention composite indicator, recent evidence consistently demonstrates that LE8 correlates with multiple health outcomes, including both CVD and non CVD diseases (13, 14). For GSD, although existing studies have analyzed the relationship between LE8 and GSD (15, 16), these investigations are limited not only by their exclusive focus on American populations but also by small sample sizes and the reliance on self-reported GSD status from study participants. In addition, it is worth noting that the potential mechanisms linking LE8 and GSD remain largely speculative at present. It is well known that inflammation and oxidative stress are important pathophysiological pathways involved in the development of many diseases, and they also influence the formation of GSD (17). Interestingly, a large body of research indicates that both the lifestyle factors and metabolic conditions included in LE8 are influences of the body's inflammatory response and oxidative stress status (18, 19). In summary, we can reasonably infer that the potential mechanism linking LE8 and GSD may be mediated by inflammation and oxidative stress. However, this remains unconfirmed by relevant studies. Therefore, leveraging baseline survey data from the China Multi-Ethnic Cohort (CMEC) study, we aim to thoroughly investigate the specific relationship between LE8 and GSD in the Chinese adult population. Furthermore, we seek to clarify whether inflammation and oxidative stress play a role in this association, ultimately providing more targeted public health interventions for preventing GSD.

## 2 Materials and methods

### 2.1 Research design and data collection

This study utilized baseline data from the CMEC to conduct a cross-sectional analysis. The CMEC is a large-scale community-based natural population cohort study conducted in Southwest China, enrolling nearly 100,000 adults aged 30–79 years. Detailed information regarding the CMEC has been published elsewhere (20). During CMEC's baseline survey, trained investigators collected data on on subjects' basic information and lifestyle behaviors. Well-trained medical personnel drew fasting blood samples from the elbow veins of study subjects and conducted standardized laboratory tests to measure biochemical indicators including fasting blood-glucose (FBG), HemoglobinA1c (HbA1c), total cholesterol (TC), and high-density lipoprotein cholesterol (HDL-C). Additionally, subjects underwent comprehensive physical examinations (including measurements of height, weight, and blood pressure) and abdominal ultrasound examination. CMEC's research design complies with the Declaration of Helsinki and has been approved by the Ethics Committee of Sichuan University (Ethics Approval Numbers: K2016038 and K2020022). Each research subject signed a written informed consent form.

**Figure 1:**
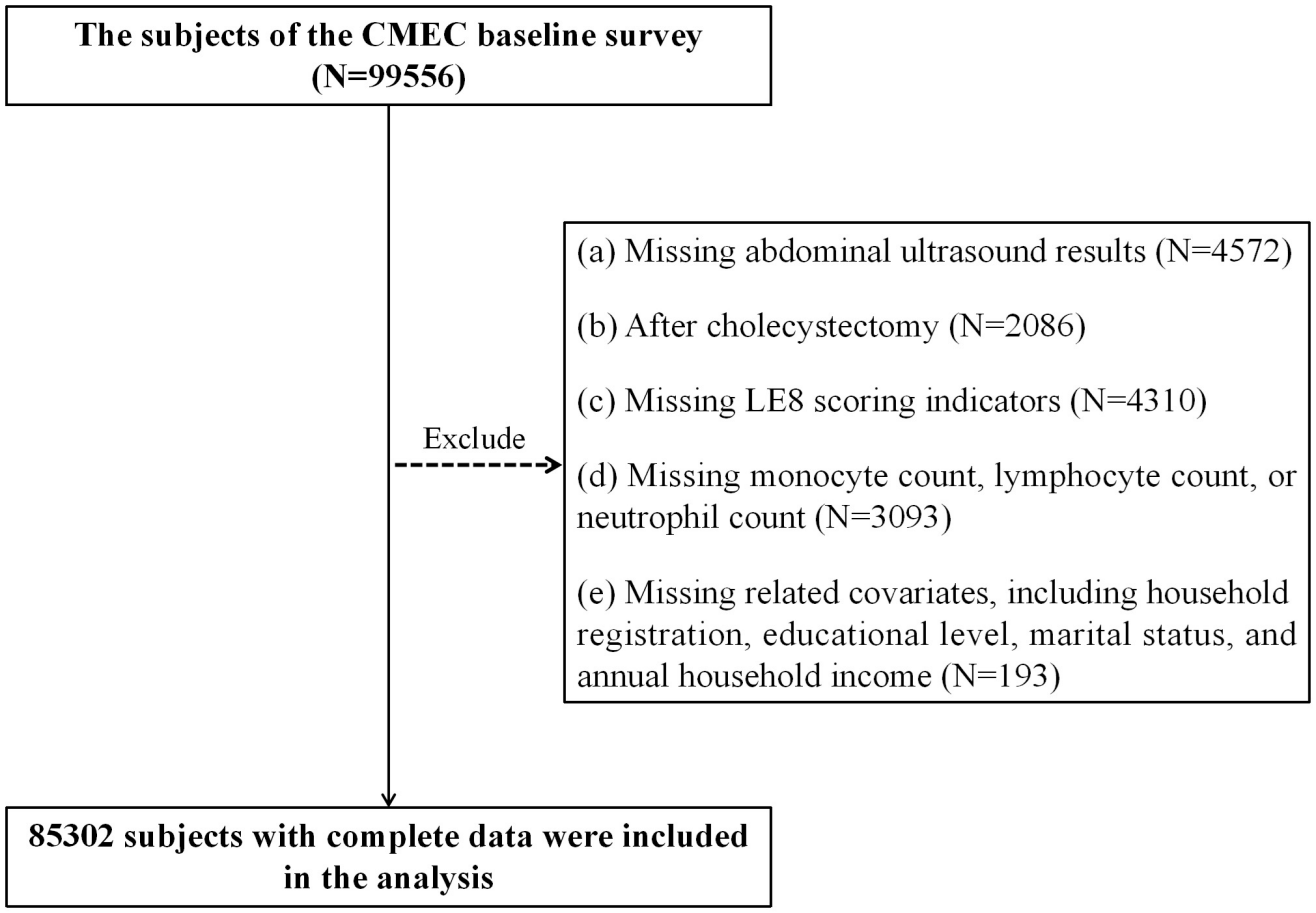
Study subject inclusion process.

As shown in Figure 1, we excluded subjects with incomplete relevant data, including those without abdominal ultrasound examination results (*n* = 4,572), those who underwent cholecystectomy (*n* = 2,086), those with missing variables required for the LE8 score (*n* = 4,310), those with missing blood cell counts (*n* = 3,093), and those with missing relevant demographic information (*n* = 193). Ultimately, 85,302 participants with complete data were included in the primary analysis.

### 2.2 Calculation of the LE8 score

The LE8 comprises eight indicators: PA, sleep, nicotine exposure, diet, BMI, blood pressure, blood glucose, and blood lipids (12). Each indicator is scored on a scale ranging from 0 to 100 points. Ultimately, the LE8 score is derived by calculating the unweighted average of the scores across eight indicators. According to the AHA recommendations, an LE8 score of 0–49 is defined as low CVH level, 50–79 as moderate CVH level, and 80–100 as high CVH level (12).

In this study, considering China's actual circumstances and cultural differences, we made appropriate adjustments to the scoring criteria for diet, PA, and BMI based on relevant research evidence. For dietary assessment, the AHA recommends using the dietary approaches to stop hypertension (DASH) scoring method to preliminarily evaluate an individual's dietary quality. This dietary pattern primarily focuses on five categories of beneficial foods recommended for increased consumption (including fresh fruits, fresh vegetables, nuts and legumes, low-fat or fat-free dairy products, and whole grains) and three categories of harmful foods advised for limited intake (including salt, red meat and its products, and sugar-sweetened beverages). In this study, since no detailed distinction was made between low-fat or fat-free dairy products, we substituted them with fish and seafood. A meta-analysis indicates that increased consumption of fish and seafood is associated with a lower risk of CVD (21). Additionally, since only 6.7% of the participants in our study population consumed sugar-sweetened beverages, we excluded this food component. In summary, we conducted a preliminary assessment of the dietary quality of study participants using the modified DASH dietary pattern (comprising seven food components: fresh fruits, fresh vegetables, whole grains, nuts and legumes, fish and seafood, sodium, and red meat and its products) (see Supplementary Table S1 in the Supplementary materials). For PA, we score based on the AHA-recommended weekly minutes of medium or high-intensity PA, combined with the metabolic equivalent of task (METs-min/week) classifications outlined in the 2024 PA Guidelines (22). For BMI, we adopted the classification criteria recommended by the AHA for Asian populations for scoring. All other indicators are scored according to the standards established by the AHA. See Supplementary Table S2 in the Supplementary material for detailed scoring standards of LE8 indicators.

### 2.3 Measurement of inflammation and oxidative stress

The systemic inflammatory response index (SIRI) was calculated to reflect the inflammatory status of the study subjects (23). The specific calculation formula is as follows (23): SIRI = (neutrophil count × monocyte count) / lymphocyte count. In this study, we divided the study population into three groups based on the SIRI tertile. Following the methodology of Li et al. (24), gamma-glutamyl transferase (GGT, log-transformed) was used as a marker for oxidative stress levels. Similarly, we grouped subjects based on the tertiles of Ln(GGT).

### 2.4 Diagnosis of GSD

Abdominal ultrasound examinations were used to diagnose GSD in participants. GSD was diagnosed when echoes dependent on gravity or ultrasound transmission attenuation (acoustic shadowing) were identified within the gallbladder or biliary tract (25). In the primary analysis of this study, participants diagnosed with cholestasis were also included in the GSD group.

### 2.5 Covariates

In this study, we considered age, gender, ethnicity, household registration status, marital status, region, education level, and annual household income as covariates. The definitions and grouping of the aforementioned variables are presented in Supplementary Table S3 in the Supplementary material.

### 2.6 Statistical methods

Continuous variables are reported as mean ± standard deviation (SD), while categorical variables are reported as frequency and percentage (%). Depending on the type of variables and the number of groups, the appropriate statistical test—*t*-test, analysis of variance, or chi-square test—be selected to compare differences between groups. Logistic regression models were employed to analyze the correlations between the LE8 score, inflammatory markers, and oxidative stress markers with GSD. Results are presented as odds ratios (*OR*) and their 95% confidence intervals (*CI*). Multiple linear regression models were employed to analyze the correlations between LE8 score and inflammatory and oxidative stress markers, with results expressed as β values and 95% *CI*. Additionally, a restricted cubic spline (RCS) with four knots at the 5th, 35th, 65th, and 95th percentiles was employed for the dose-response analysis between LE8 score and GSD. In the multivariate analysis, we established three adjustment models to progressively adjust for confounding factors. Model 1 was not adjusted for any covariates. Model 2 was adjusted for age and gender. Model 3 was further adjusted for ethnicity, household registration, marital status, region, education level, and annual household income based on Model 2. We used the “mediation” package in R to analyze the mediating effects of inflammation and oxidative stress between LE8 and GSD, and assessed the 95% *CI* for the proportion of mediation using a bootstrap method with 5,000 iterations. Additionally, subgroup and interaction analyses were conducted to assess the specific effects of LE8 on GSD across different populations. Finally, we conducted two sensitivity analyses to assess the robustness of our findings by: (1) excluding subjects with self-reported history of CVD, and (2) further excluding subjects with cholestasis. Excel 2010, SPSS 26, and R Studio were used for Arrangement and analysis of data. Statistical tests were two-tailed. Results were considered statistically significant when *P* < 0.05.

## 3 Results

### 3.1 Basic characteristics of the study population

This study included a total of 85,302 eligible subjects with an average age of 51.56 ± 11.55 years, of whom 39.90% were male. Ultimately, a total of 6,197 participants (7.26%) were diagnosed with GSD. Stratified by GSD status, the basic characteristics are summarized in Table 1. For demographic characteristics, the GSD group exhibited a higher average age. Furthermore, the prevalence of GSD was significantly elevated among females, ethnic minorities, urban residents, single-person households, and individuals with higher annual household income (all *P* < 0.001). In terms of inflammation, oxidative stress, and LE8 score, the GSD group exhibited significantly higher SIRI index and Ln(GGT) level compared to the non GSD group, while demonstrating lower LE8 score (all *P* < 0.001). When grouped by CVH levels (Supplementary Table S4), the SIRI index, Ln(GGT), and GSD prevalence all decreased significantly with increasing CVH levels (all *P* < 0.001).

**Table 1:** Basic characteristics of the study population.

Characteristics	General population	Non GSD group	GSD group	*P*-value
	*N* = 85,302	*N* = 79,105	*N* = 6,197	
Age group, *N* (%)				
< 50	39,422 (46.21)	37,146 (94.23)	2,276 (5.77)	< 0.001
≥50	45,880 (53.79)	41,959 (91.45)	3,921 (8.55)	
Gender, *N* (%)				
Male	34,036 (39.90)	31,780 (93.37)	2,256 (6.63)	< 0.001
Female	51 266 (60.10)	47,325 (92.31)	3,941 (7.69)	
Ethnicity, *N* (%)				
Han ethnicity	50,958 (59.74)	47,588 (93.39)	3,370 (6.61)	< 0.001
Ethnic minorities^a^	34,344 (40.26)	31,517 (91.77)	2,827 (8.23)	
Household Registration, *N* (%)				
Rural	55,916 (65.55)	52,253 (93.45)	3,663 (6.55)	< 0.001
Urban	29,386 (34.45)	26,852 (91.38)	2,534 (8.62)	
Marital status, *N* (%)				
Married and living together	75,682 (88.72)	70,318 (92.91)	5,364 (7.09)	< 0.001
Single^b^	9,620 (11.28)	8,787 (91.34)	8,33 (8.66)	
Region, *N* (%)				
Sichuan basin	41,046 (48.12)	38,119 (92.87)	2,927 (7.13)	0.345
Yungui plateau	38,166 (44.74)	35,349 (92.62)	2,817 (7.38)	
Qinghai-Tibet plateau	6,090 (7.14)	5,637 (92.56)	4,53 (7.44)	
Education level, *N* (%)				
Elementary school and below	43,402 (50.88)	40,305 (92.86)	3,097 (7.14)	0.143
Junior high school and high school	32,318 (37.89)	29,956 (92.69)	2,362 (7.31)	
Associate degree or higher	9,582 (11.23)	8,844 (92.30)	7,38 (7.70)	
Annual household income (¥), *N* (%)				
< 20,000	30,541 (35.80)	28,403 (93.00)	2,138 (7.00)	0.002
20,000–100,000	43,320 (50.78)	40,175 (92.74)	3,145 (7.26)	
>100,000	11,441 (13.41)	10,527 (92.01)	9,14 (7.99)	
Age (years), mean ± SD	51.56 ± 11.55	51.36 ± 11.52	54.10 ± 11.52	< 0.001
Monocyte count (10^9^/L), mean ± SD	0.35 ± 0.15	0.34 ± 0.15	0.36 ± 0.15	< 0.001
Lymphocyte count (10^9^/L), mean ± SD	1.82 ± 0.69	1.82 ± 0.69	1.84 ± 0.70	0.148
Neutrophil count (10^9^/L), mean ± SD	3.78 ± 1.42	3.77 ± 1.43	3.87 ± 1.39	< 0.001
SIRI index, mean ± SD	0.80 ± 0.62	0.79 ± 0.62	0.85 ± 0.62	< 0.001
Ln(GGT), mean ± SD	3.28 ± 0.74	3.27 ± 0.73	3.42 ± 0.77	< 0.001
Diet score, mean ± SD	35.67 ± 31.54	35.70 ± 31.54	35.36 ± 31.51	0.418
Sleep score, mean ± SD	81.92 ± 26.58	81.98 ± 26.56	81.27 ± 26.78	0.044
Nicotine exposure score, mean ± SD	70.44 ± 37.59	70.29 ± 37.68	72.44 ± 36.37	< 0.001
PA score, mean ± SD	46.04 ± 23.57	46.38 ± 23.62	41.65 ± 22.47	< 0.001
BMI score, mean ± SD	73.84 ± 24.30	74.36 ± 24.18	67.17 ± 24.75	< 0.001
Blood lipid score, mean ± SD	71.51 ± 29.38	71.77 ± 29.35	68.11 ± 29.66	< 0.001
Blood glucose score, mean ± SD	78.59 ± 24.74	78.85 ± 24.57	75.24 ± 26.54	< 0.001
Blood pressure score, mean ± SD	59.85 ± 35.35	60.31 ± 35.27	53.89 ± 35.76	< 0.001
LE8 score, mean ± SD	64.73 ± 12.85	64.95 ± 12.85	61.89 ± 12.62	< 0.001

“^a^” refers to the Tibetan, Yi, Miao, Bai, Dong, and Buyi ethnic groups. “^b^” refers to marital status including widowed, divorced, separated, and never married. GSD, gallstone disease; SIRI, systemic inflammatory response index; GGT, gamma-glutamyl transferase; PA, Physical activity; BMI, body mass index; LE8, life's essential 8, SD, standard deviation.

### 3.2 Correlation between LE8 and GSD

Table 2 presents the results of the multivariate logistic regression analysis examining the relationship between LE8 score and GSD. After adjusting for age, gender, ethnicity, household registration, marital status, region, education level, and annual household income, the results revealed a significant negative correlation between LE8 score and GSD (For each 10-point increase in LE8: *OR* = 0.833, 95% *CI*: 0.815 to 0.852, *P* < 0.001). When grouped by CVH levels, the risk of GSD was reduced by 25.6 and 53.3% in the moderate (*OR* = 0.744, 95% *CI*: 0.692–0.801, *P* < 0.001) and high (*OR* = 0.467, 95% *CI*: 0.415–0.526, *P* < 0.001) CVH groups, respectively, compared to the low CVH group. Further analysis of the correlation between LE8 component indicators and GSD revealed significant negative correlations between PA, BMI, blood pressure, blood glucose, and blood lipids with GSD (all *P* < 0.001). However, no statistically significant associations were found between diet, sleep, and nicotine exposure with GSD (Supplementary Table S5).

**Table 2:** Multivariate logistic regression analysis results for LE8 score and GSD.

LE8 score	Model 1		Model 2		Model 3	
	OR (95%CI)	*P*-value	OR (95%CI)	*P*-value	OR (95%CI)	*P*-value
For each 10-point increase in LE8	0.840 (0.824, 0.857)	< 0.001	0.830 (0.812, 0.848)	< 0.001	0.833 (0.815, 0.852)	< 0.001
Classified						
Low CVH	Ref.		Ref.		Ref.	
Moderate CVH	0.744 (0.694, 0.798)	< 0.001	0.730 (0.679, 0.785)	< 0.001	0.744 (0.692, 0.801)	< 0.001
High CVH	0.446 (0.400, 0.497)	< 0.001	0.457 (0.407, 0.514)	< 0.001	0.467 (0.415, 0.526)	< 0.001

Model1: no covariates adjusted; Model 2: adjusted for age and gender; Model 3: Adjusted for age, gender, ethnicity, household registration, marital status, region, education level, and annual household income. LE8, life's essential 8; CVH, cardiovascular health; GSD, gallstone disease; OR, odds ratio; CI, confidence interval.

### 3.3 Dose-response relationship between LE8 score and GSD

**Figure 2:**
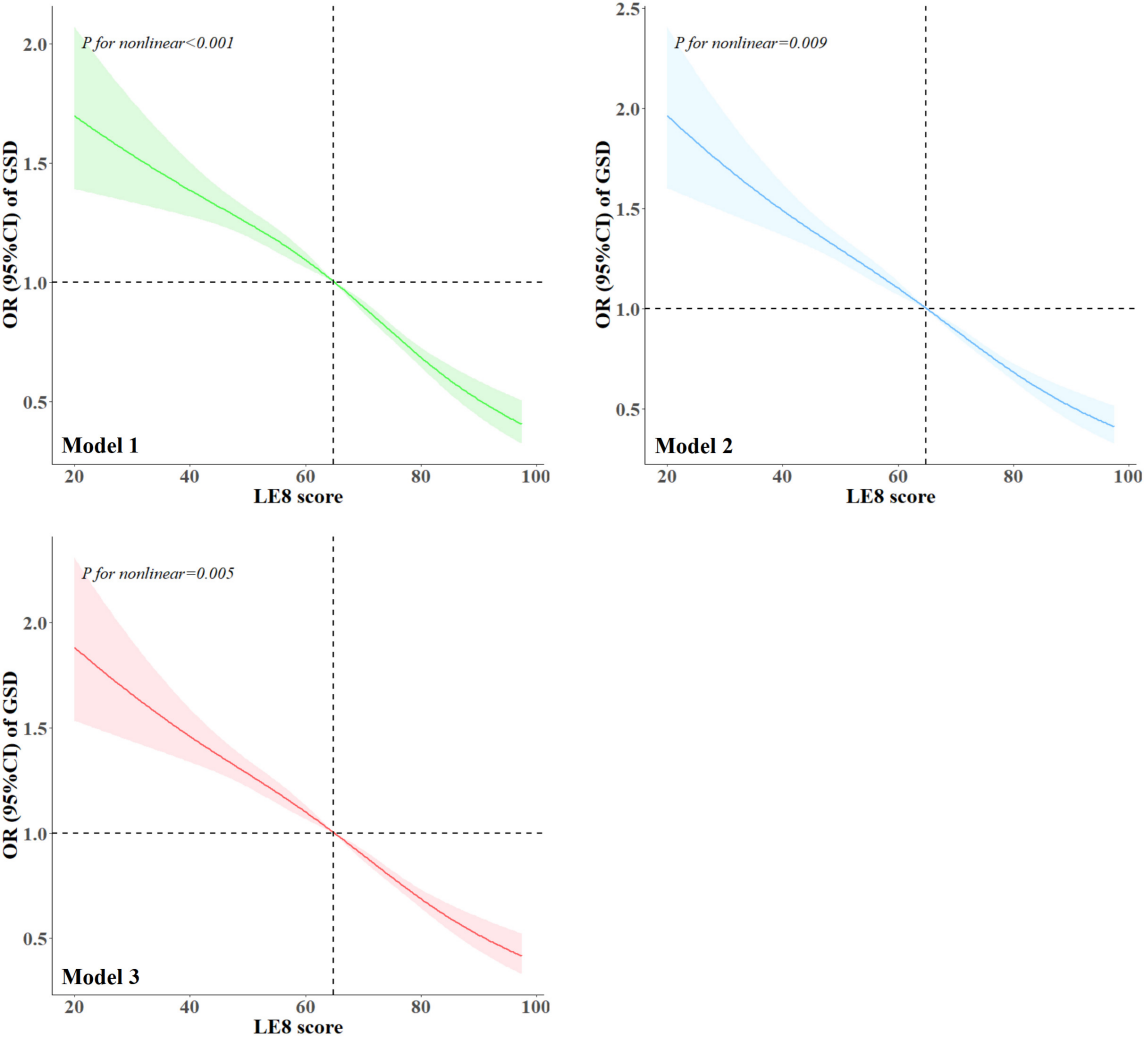
Dose-response relationship between LE8 and GSD. The solid line represents the fitted smooth curve between variables, and the shaded area indicates the fitted 95% confidence interval. Model1: no covariates adjusted; Model 2: adjusted for age and gender; Model 3: adjusted for age, gender, ethnicity, household registration, marital status, region, education level, and annual household income. LE8, life's essential 8; GSD, gallstone disease; OR, odds ratio; CI, confidence interval.

Figure 2 presents the results of the RCS regression analysis between LE8 score and GSD. The findings indicate a significant nonlinear inverse dose-response relationship between LE8 score and GSD (*P* for nonlinear = 0.005). Specifically, as the LE8 score increases, the overall risk of GSD shows a decreasing trend, but this decline exhibits varying rates across different score intervals. This is manifested as a steeper rate of reduction in GSD risk per unit increase in LE8 score in the higher score interval (LE8 score > 64.79) compared to the lower score interval.

### 3.4 Association between LE8 and inflammation and oxidative stress

In the fully adjusted model, the LE8 score showed significant negative correlations with both SIRI and Ln(GGT) (all *P* < 0.001). Specifically, for each 10-point increase in LE8, the β value for SIRI and its 95% *CI* was −0.023 (−0.027–−0.020), while the β value for Ln(GGT) and its 95% *CI* was −0.174 (−0.178–−0.171). Detailed results are summarized in Table 3.

**Table 3:** Results of multivariate linear regression analysis between LE8 and inflammation and oxidative stress.

Independent variable	SIRI		Ln(GGT)	
	β (95%CI)^*^	*P*-value	β (95%CI)^*^	*P*-value
For each 10-point increase in LE8	−0.023 (−0.027, −0.020)	< 0.001	−0.174 (−0.178, −0.171)	< 0.001
Classified				
Low CVH	Ref.		Ref.	
Moderate CVH	−0.082 (−0.094, −0.069)	< 0.001	−0.369 (−0.382, −0.355)	< 0.001
High CVH	−0.092 (−0.109, −0.075)	< 0.001	−0.674 (−0.693, −0.655)	< 0.001

“^*^” indicates adjustment for age, gender, ethnicity, household registration, marital status, region, education level, and annual household income. LE8, life's essential 8; SIRI, systemic inflammatory response index; GGT, gamma-glutamyl transferase; CI, confidence interval.

### 3.5 The association between inflammation, oxidative stress, and GSD

Table 4 summarizes the results of correlation analyses between inflammatory and oxidative stress biomarkers and GSD. In the fully adjusted model, both SIRI and Ln(GGT) showed significant positive correlations with GSD (all *P* < 0.001). In other words, higher levels of SIRI and Ln(GGT) are associated with an increased risk of GSD.

**Table 4:** Results of multivariate logistic regression analysis between SIRI, Ln(GGT), and GSD.

Independent variable	OR (95%CI)^*^	*P*-value
Inflammatory biomarker, SIRI		
Continuous	1.134 (1.093, 1.175)	< 0.001
Classified		
First tertile	Ref.	
Second tertile	1.148 (1.074, 1.226)	< 0.001
Third tertile	1.331 (1.245, 1.422)	< 0.001
Oxidative stress biomarker, Ln(GGT)		
Continuous	1.386 (1.338, 1.436)	< 0.001
Classified		
First tertile	Ref.	
Second tertile	1.393 (1.299, 1.493)	< 0.001
Third tertile	1.884 (1.755, 2.022)	< 0.001

“^*^” indicates adjustment for age, gender, ethnicity, household registration, marital status, region, education level, and annual household income. SIRI, systemic inflammatory response index; GGT, gamma-glutamyl transferase; GSD, gallstone disease; OR, odds ratio; CI, confidence interval.

### 3.6 Mediating role of inflammation and oxidative stress between LE8 and GSD

**Figure 3:**
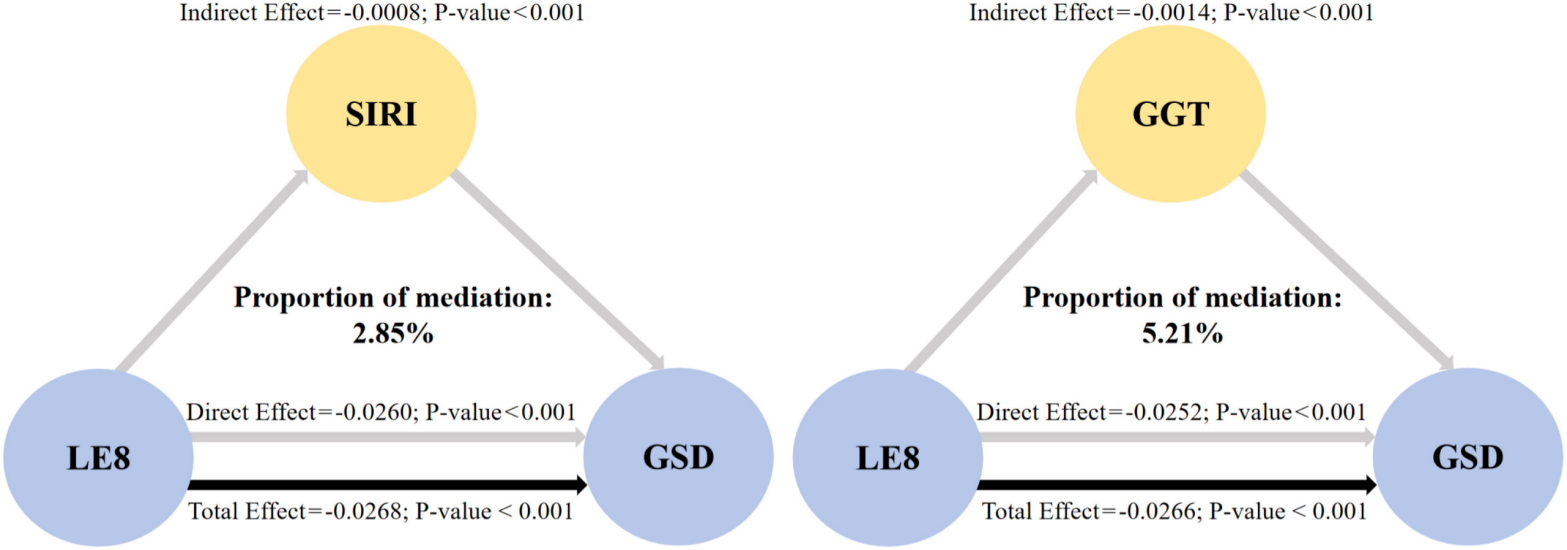
Pathway analysis of the mediating effects of inflammation and oxidative stress between LE8 and GSD. For each 10-point increase in LE8 as the independent variable, GSD serves as the dependent variable, with SIRI and GGT acting as mediating variables. The total effect represents the overall relationship between LE8 and GSD that is unaffected by mediating variables. The direct effect indicates the direct relationship between LE8 and GSD after controlling for SIRI or GGT. The indirect effect reflects the influence of LE8 on GSD mediated through SIRI or GGT. LE8, life's essential 8; SIRI, systemic inflammatory response index; GGT, gamma-glutamyl transferase; GSD, gallstone disease.

To verify whether inflammation and oxidative stress mediate the association between LE8 and GSD, we conducted a mediation analysis with LE8 (for each 10-point increase) as the independent variable, GSD as the dependent variable, and SIRI and GGT as mediating variables. As shown in Figure 3, LE8 exerted a significant indirect effect on GSD through SIRI, with an indirect effect size of −0.0008 (95% *CI*: −0.0010–−0.0005, *P* < 0.001). Similarly, LE8 exerted a significant indirect effect on GSD via GGT, with an indirect effect size of −0.0014 (95% *CI*: −0.0019–−0.0009, *P* < 0.001). The results indicate that both inflammation and oxidative stress significantly mediate the relationship between LE8 and GSD. The mediation proportions were 2.85% for SIRI and 5.21% for GGT (Table 5).

**Table 5:** Results of mediator effect analysis for inflammation and oxidative stress between LE8 and GSD.

Independent variable	Mediating variable	Total effect	Direct effect	Indirect effects	Proportion of mediation
		Coefficient (95%CI)	Coefficient (95%CI)	Coefficient (95%CI)	
For each 10-point increase in LE8	SIRI	−0.0268 (−0.0317, −0.0220)	−0.0260 (-0.0310,−0.0213)	−0.0008 (-0.0010,−0.0005)	2.85%
For each 10-point increase in LE8	GGT	−0.0266 (−0.0316, −0.0219)	−0.0252 (−0.0301, −0.0206)	−0.0014 (−0.0019, −0.0009)	5.21%

LE8, life's essential 8; SIRI, systemic inflammatory response index; GGT, gamma-glutamyl transferase; GSD, gallstone disease; CI, confidence interval.

### 3.7 Subgroup and interaction analysis

**Figure 4:**
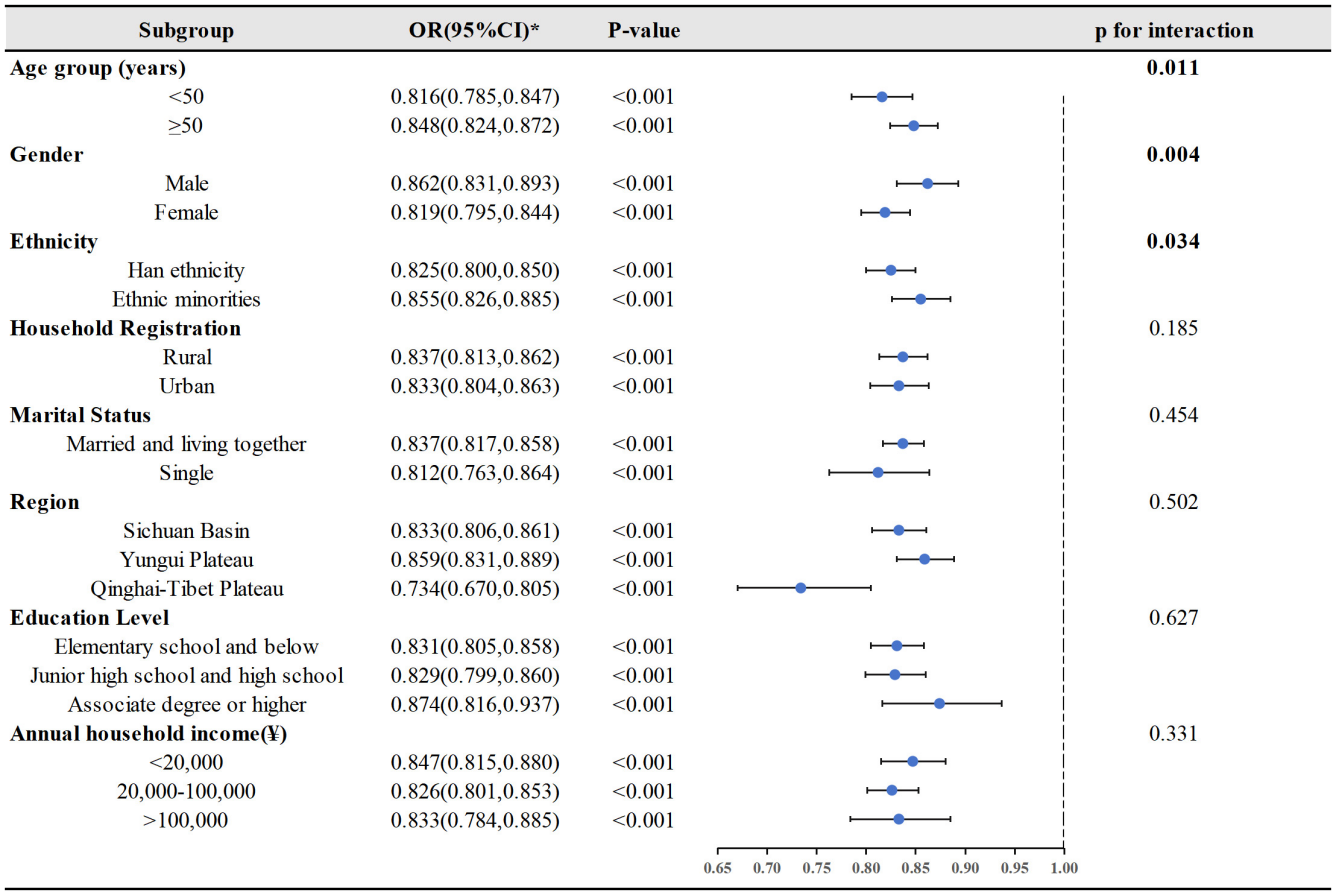
Results of subgroup and interaction analyses between LE8 score and GSD. Adjustments were made for age, gender, ethnicity, household registration, marital status, region, education level, and annual household income. LE8, life's essential 8; GSD, gallstone disease; OR, odds ratio; CI, confidence interval.

Figure 4 illustrates the correlation between LE8 and GSD across different demographic subgroups. Across all subgroups, LE8 exhibited a significant negative correlation with GSD (all *P* < 0.001). Notably, further interaction analysis revealed significant interactions of LE8 with age, gender, and ethnicity (all *P* for interaction < 0.05). Specifically, the negative correlation between LE8 score and GSD was more pronounced among participants who were under 50 years old, female, or of Han ethnicity.

### 3.8 Sensitivity analysis

The results of two sensitivity analyses show that the OR values of LE8 and GSD are almost unchanged (Supplementary Table S6), with the dose-response relationship also demonstrating nonlinearity (Supplementary Figure S1). In the mediation analysis, the mediation effect of inflammation and oxidative stress is still significant, especially after excluding the subjects with CVD history and cholestasis, the proportion of mediation increases slightly (Supplementary Tables S7, S8, Supplementary Figure S2).

## 4 Discussion

This cross-sectional analysis of 85,302 adults in Southwest China demonstrated a significant nonlinear inverse relationship between LE8 score and the risk of GSD. When categorizing CVH levels based on the AHA-recommended LE8 cutoff values, the risk of GSD was reduced by 25.6 and 53.3% in moderate and high CVH levels, respectively, compared to low CVH level. Furthermore, mediation analysis revealed that this inverse association of LE8-GSD was partially mediated by inflammation and oxidative stress.

To our knowledge, previous studies have evaluated the relationship between the LE8 score and GSD. It is worth noting that previous studies have found only high CVH level to be associated with a reduced risk of GSD, while moderate CVH level showed no significant association (15, 16), which is inconsistent with our findings. This discrepancy may be related to the diagnostic methods for GSD. In the early stages of GSD, approximately 75% of patients exhibit no symptoms or discomfort whatsoever (9). Consequently, these asymptomatic early-stage GSD patients are often only incidentally detected through imaging examinations (2). Previous studies have relied on self-reported GSD status among participants (15, 16), which may not avoid misclassification bias, thereby underestimating the true association between CVH levels and GSD. According to relevant research reports, experienced ultrasound specialists can achieve a sensitivity of up to 95% and a specificity approaching 100% when detecting GSD through abdominal ultrasound examinations (25). In our study, all GSD diagnoses were confirmed by specialized sonographers through abdominal ultrasound examinations, significantly enhancing diagnostic accuracy and thereby strengthening the reliability of our findings.

It is well known that altering habitual behaviors in individuals is a complex process. Our findings indicate that not only high CVH level, but even moderate CVH level can significantly reduce the risk of GSD. This discovery may hold important implications for public health practice, particularly for populations with low CVH level. For them, setting a relatively easier and more achievable goal may prove more motivating and sustainable than simply advocating for an unrealistic “high standard” objective. The Health Belief Model indicates that perceived risk of disease is a crucial prerequisite for health behavior change (26). However, a meta-analysis further emphasizes that enhancing individuals' self-efficacy and perceived benefits are key drivers for sustaining action and effort (27). In this regard, setting an achievable action goal helps lower the “psychological barrier” in behavior change, thereby enhancing self-efficacy (28). Consequently, our study provides scientific evidence for public health practitioners and clinicians to advocate for progressive, achievable LE8 health promotion strategies when guiding patients or high-risk populations in preventing GSD. However, it should be noted that our dose-response analysis revealed a more pronounced reduction in GSD risk for each incremental increase in LE8 score once the score reached higher levels. This suggests that maintaining and pursuing higher LE8 score remains a long-term goal for GSD prevention.

Currently, the specific pathway linking LE8 and GSD remains unclear. Our study is the first to reveal, through mediation analysis, that inflammation and oxidative stress significantly mediate the relationship between LE8 and GSD. This suggests that the healthy lifestyle and physiological metabolic state promoted by LE8 may partially reduce the risk of GSD by alleviating or improving chronic inflammation levels and oxidative damage in the body. It is well known that inflammation and oxidative stress are two closely linked and interdependent pathophysiological processes (19) that play significant roles in the onset and progression of numerous diseases, including GSD (17). Research indicates that the activation of inflammasomes, such as NOD-like receptor protein 3, participates in cholesterol crystal-induced mucus secretion, thereby promoting gallstone formation (29). Additionally, inflammatory mediators such as tumor necrosis factor-alpha can induce increased remodeling of bile duct epithelial cells and promote heightened mucoprotein expression. This ultimately leads to cholestasis and reduced gallbladder motility (30), both of which are significant contributing factors to gallstone formation. Another animal study found that activating specific pathways that suppress inflammatory responses in the body significantly reduced the risk of gallstone formation in mice fed a high-fat diet (31). For oxidative stress, a high level of oxidative stress has been shown to cause damage to gallbladder mucosal cells, leading to abnormalities in the gallbladder's absorption and secretory functions and promoting the formation of gallstones (32). In this study, consistent with previous findings (33), we observed that biomarkers associated with inflammation and oxidative stress both exhibited a significant positive correlation with GSD risk. This further confirms in natural populations that inflammation and oxidative stress constitute a critical pathological microenvironment directly involved in GSD development.

Although inflammation and oxidative stress are key contributors to the pathogenesis of GSD, extensive research indicates that various lifestyle behaviors and biological factors significantly influence inflammatory and oxidative stress states (18, 19). For example, a healthy diet not only directly reduces pro-inflammatory substances and increases antioxidant intake but also indirectly enhances the body's defense against inflammation and oxidative stress by optimizing the gut microbiota (19, 34). Similarly, regular physical exercise not only directly reduces systemic inflammation and oxidative stress but also indirectly exerts anti-inflammatory and antioxidant effects by improving the function of adipose tissue and skeletal muscle (35). In the process by which smoking causes numerous chronic diseases, inflammation and oxidative stress are also considered important pathological foundations (36). A recent study indicates that smokers can improve skeletal muscle resistance to fatigue even 2 weeks after quitting, thereby reducing inflammatory damage (37). Regarding sleep, the latest research evidence indicates that sleep deprivation can suppress melatonin secretion, thereby promoting the occurrence of inflammation and oxidative stress (38). In obese states, adipocyte dysfunction can activate pro-inflammatory transcription factors, leading to the release of inflammatory mediators such as tumor necrosis factor-alpha, interleukin-1β, and interleukin-6. In turn, these inflammatory mediators further stimulate the production and accumulation of reactive oxygen species (39). Therefore, scientifically reducing body weight helps lower levels of inflammation and oxidative stress. It is worth noting that a population-based intervention study found that a comprehensive intervention combining multiple lifestyle factors yields greater effects in improving inflammation and oxidative stress than single-factor interventions alone (40). In our study, increasing the LE8 score—which integrates multiple lifestyle factors and physiological metabolic conditions—significantly reduced levels of inflammation and oxidative stress, further supporting prior research evidence.

It is important to note that the mediation analysis in this study revealed that SIRI and GGT mediated 2.85 and 5.21% of the association between LE8 and GSD, respectively. This suggests that there are other, as-yet-unmeasured mechanisms underlying the protective effect of LE8 against GSD. For example, potential mediators such as the gut microbiota and its metabolites, adipokines, and bile acids may be involved (41, 42). Future studies should employ multi-omics technologies, such as metagenomics, metabolomics, and proteomics, to systematically screen for these potential mediators, thereby providing a more comprehensive understanding of the networked mechanisms through which LE8 reduces the risk of GSD. Nevertheless, by linking LE8 to GSD via inflammatory and oxidative stress pathways, this study provides an important pathophysiological foundation for comprehensive public health interventions targeting GSD.

In the interaction analysis, we found significant age, gender, and ethnic differences in the strength of the inverse association between LE8 and GSD. Regarding age, one study indicates that the risk of developing GSD increases significantly with advancing age (43). This is believed to be associated with declining gallbladder function that occurs with aging. In our study, the preventive effect of LE8 against GSD was more pronounced in younger participants than in older ones, consistent with findings from Wang et al. (15). Furthermore, a prospective cohort study similarly demonstrated that accumulating higher LE8 scores during adolescence yields more significant effects on reducing CVD incidence and mortality risk in middle age and beyond (13). Collectively, these studies underscore that improving and maintaining higher LE8 levels during adolescence holds greater practical significance for future health outcomes. It is worth noting that, compared to previous studies, we also found that intervention with LE8 can significantly improve women's inherent high risk of GSD. All epidemiological studies to date consistently demonstrate that the incidence and prevalence of GSD are significantly higher in females than in males (43, 44). This gender difference is typically attributed to estrogen's role in promoting cholesterol synthesis and crystallization in the liver, thereby increasing the risk of stone formation in women (45). However, our research indicates that women demonstrate a more pronounced effect in preventing GSD by improving their LE8 score compared to men. This suggests that public health interventions and clinical guidance should place greater emphasis on the critical importance of healthy lifestyles for women in preventing GSD. Additionally, we observed that the protective association between LE8 and GSD was more pronounced in the the Han ethnicity population compared to ethnic minority groups. As previously mentioned, the etiology of GSD is complex, resulting from the interaction between genetic and modifiable environmental factors (9). Multiple studies have demonstrated significant racial disparities in the incidence and prevalence of GSD (46, 47). In China, Lv et al. found that the prevalence of GSD among Uyghurs and other ethnic minorities was also significantly higher than that among the Han ethnicity (46). They pointed out that, in addition to some traditional risk factors, this disparity may also be related to the living environments, specific cultural practices, and genetic factors among ethnic minority populations. Therefore, we speculate that ethnic minority populations may possess additional risk factors beyond LE8, thereby partially offsetting the benefits conferred by LE8.

Our study utilized a natural population sample of over 80,000 subjects, with GSD diagnoses confirmed through abdominal ultrasound examinations performed by specialized sonographers. These strengths enhance the representativeness and accuracy of our findings. Furthermore, we not only identified significant age-, gender-, and ethnicity-specific variations in the association between LE8 and GSD, but also revealed that the protective association between the two is partially mediated by inflammation and oxidative stress. These new findings will help provide a more comprehensive public health perspective for developing targeted interventions for GSD. Nevertheless, this study has several limitations that warrant consideration. First, as a cross-sectional study, it cannot clarify the temporal relationship between the two events and therefore cannot infer a causal relationship between them. Although we observed a negative correlation between the LE8 score and the risk of GSD, the possibility of a reverse association cannot be ruled out. For example, patients with GSD may alter their lifestyle due to pain or digestive discomfort, thereby affecting the LE8 score. Therefore, future studies should employ intervention studies or prospective cohort studies to further establish a causal relationship between LE8 and GSD. Second, regarding biomarkers of inflammation and oxidative stress, while SIRI and GGT are suitable choices for large population cohorts, it must be acknowledged that they are merely general systemic markers. Therefore, we recommend that future studies (particularly prospective or intervention studies) further investigate more specific molecular biomarkers (such as C-reactive protein, interleukins, or malondialdehyde) to validate these mechanistic pathways. Third, data on lifestyle behaviors and other aspects were collected through questionnaires. Although we provided standardized training to the survey staff prior to the investigation, it was still impossible to completely eliminate the influence of recall bias. In addition, subjects with incomplete data were excluded from the analysis; this exclusion may not have been entirely random, which could introduce selection bias. Fourth, although we controlled for certain confounding factors in our statistical analysis, residual confounding cannot be ruled out as a potential influence on the results. Fifth, the participants in this study were exclusively adults aged 30–79 from Southwest China. Therefore, the generalizability of the findings may be limited, particularly for the specific population of children and adolescents. Sixth, due to the lack of data on genetic risk factors for GSD, this study was unable to further analyze whether genetic factors influence the specific association between LE8 and GSD, or whether they affect the mediating role of inflammation and oxidative stress. Future research should delve deeper into these areas, which will help provide personalized evidence for GSD prevention. Finally, different types of gallstones—such as cholesterol stones, brown pigment stones, and melanin stones—have distinct etiologies and pathogenesis. This study employed abdominal ultrasound, which cannot differentiate the specific type of gallstones in GSD patients, potentially influencing the research findings.

## 5 Conclusion

Among Chinese adults, maintaining higher LE8 score is associated with a lower risk of GSD. This inverse relationship is partially mediated through improvements in systemic inflammation and oxidative stress levels. The preventive effect of increasing LE8 score is more pronounced in younger individuals, women, and the Han ethnicity. These findings provide new public health perspectives for developing targeted interventions against GSD.

## Data Availability

The raw data supporting the conclusions of this article will be made available by the authors, without undue reservation.
