# Supplementary material for: Better adherence to the life's essential 8 can reduce the risk of gallstone disease: mediated by inflammation and oxidative stress
Source: Front Public Health. 2026 Jun 4;14:1836977. doi: 10.3389/fpubh.2026.1836977 (PMC13275442; doi:10.3389/fpubh.2026.1836977)
Supplement: Supplementary file 1 [file Table_1.docx]

**Better adherence to the life’s essential 8 can reduce the risk of gallstone disease: Mediated by Inflammation and Oxidative Stress**

**Content**

Table S1: Food components and scoring criteria for the modified DASH dietary pattern

Table S2: Component indicators and scoring criteria for the Life’s Essential 8

Table S3: Definition and grouping of relevant covariates

Table S4: Basic characteristics of study population grouped by CVH level

Table S5: Correlation analysis between LE8 series indicator score and GSD

Table S6: Sensitivity analysis of the relationship between LE8 and GSD

Table S7: Sensitivity analysis of the mediating effect of SIRI on the relationship between LE8 and GSD

Table S8: Sensitivity analysis of the mediating effect of GGT on the relationship between LE8 and GSD

Figure S1: Sensitivity analysis of the dose-response relationship between LE8 and GSD risk

Figure S2: Sensitivity analysis of the mediating effect pathways of inflammation and oxidative stress between LE8 and GSD

Table S1: Food components and scoring criteria for the modified DASH dietary pattern

| Food categories | Scoring Criteria | lowest level | highest level |
| --- | --- | --- | --- |
|  | Positive scoring |  |  |
| fresh fruits | Q1 = 1 point | Q1 | Q5 |
| fresh vegetables | Q2 = 2 points | Q1 | Q5 |
| whole grains | Q3 = 3 points | Q1 | Q5 |
| nuts and legumes | Q4 = 4 points | Q1 | Q5 |
| fish and seafood | Q5 = 5 points | Q1 | Q5 |
|  | Reverse scoring |  |  |
| red meat and its products | Q1 = 5 points | Q5 | Q1 |
| sodium | Q2 = 4 points | Q5 | Q1 |
|  | Q3 = 3 points |  |  |
|  | Q4 = 2 points |  |  |
|  | Q5 = 1 points |  |  |

Note: Q denotes quintiles, where Q1 represents the first quintile, Q2 the second quintile, and so on. Specifically, we assigned positive scores to fresh fruits, fresh vegetables, whole grains, nuts and legumes, and fish and seafood based on the quintiles of intake for each food group in the study population (i.e., Q1 receives 1 point, Q5 receives 5 points). For salt, red meat, and processed meat, we assign negative scores (i.e., Q1 receives 5 points, while Q5 receives 1 point). Finally, by summing the scores for each food group, we obtain a modified DASH diet score ranging from 7 to 35 points.

Table S2: Component indicators and scoring criteria for the Life’s Essential 8

| Indicator | Quantification and scoring criteria | |
| --- | --- | --- |
| PA | Metabolic equivalent of task (METs-min/week) | Score |
|  | ≥500 | 100 |
|  | ≥400 | 90 |
|  | ≥300 | 80 |
|  | ≥200 | 60 |
|  | ≥100 | 40 |
|  | ≥3.33 | 20 |
|  | ≥0 | 0 |
| Nicotine Exposure | Tobacco use status | Score ^a^ |
|  | Never smoking | 100 |
|  | Previously smoked, quit smoking ≥5 years | 75 |
|  | Previously smoked, quit smoking 1-5 years ago | 50 |
|  | Previously smoked, quit smoking <1 year | 25 |
|  | Smoking | 0 |
| Sleep (hours/night) | Average sleep duration per night | Score |
|  | 7- <9 | 100 |
|  | 9- <10 | 90 |
|  | 1. < 7 | 70 |
|  | <6 or ≥ 10 | 40 |
|  | < 5 | 20 |
|  | < 4 | 0 |
| Diet | the Modified DASH dietary pattern score | Score |
|  | ≥ 95th | 100 |
|  | 75th - 94th | 80 |
|  | 50th - 74th | 50 |
|  | 25th - 49th | 25 |
|  | 1st - 24th | 0 |
| BMI (kg/m2) | Weight (kg) ÷ Height (m) squared | Score |
|  | ≤ 22.9 | 100 |
|  | 23.0 - 24.9 | 75 |
|  | 25.0 - 29.9 | 50 |
|  | 30.0 - 34.9 | 25 |
|  | ≥ 35.0 | 0 |
| Blood Lipid (mg/dl) | Non HDL-C = TC − HDL-C | Score ^b^ |
|  | <130 | 100 |
|  | 130 - 159 | 60 |
|  | 160 - 189 | 40 |
|  | 190 - 219 | 20 |
|  | ≥ 220 | 0 |
| Blood Glucose | FBG (mg/dl) or HbA1c (%) | Score |
|  | FBG < 100 (or HbA1c < 5.7) | 100 |
|  | FBG 100 - 125 (or HbA1c 5.7 - 6.4) | 60 |
|  | HbA1c < 7.0 | 40 |
|  | HbA1c 7.0 - 7.9 | 30 |
|  | HbA1c 8.0 - 8.9 | 20 |
|  | HbA1c 9.0 - 9.9 | 10 |
|  | HbA1c ≥ 10.0 | 0 |
| Blood Pressure (mmHg) | Systolic blood pressure and diastolic blood pressure | Score ^b^ |
|  | < 120 / < 80 | 100 |
|  | 120 - 129 / < 80 | 75 |
|  | 130 - 139 or 80 - 89 | 50 |
|  | 140 - 159 or 90 - 99 | 25 |
|  | ≥ 160 or ≥ 100 | 0 |

Note: “a” indicates that if the research subject self-reports having active indoor smoker in the household, the score should be reduced by 20 points (excluding 0 points); “b” indicates that if the level is post-treatment, the score should be reduced by 20 points (excluding 0 points). Abbreviation: PA, Physical activity; DASH, The Dietary Approaches to Stop Hypertension; BMI, body mass index; TC, Total Cholesterol; HDL-C, High-Density Lipoprotein Cholesterol; non HDL-C, Non-High Density Lipoprotein Cholesterol; FBG, fasting blood-glucose; HbA1c, Hemoglobin A1c。

Table S3: Definition and grouping of relevant covariates

| Variable | Grouping |
| --- | --- |
| Age (years) | <50; ≥50 |
| Gender | Male; Female |
| Ethnicity | Han ethnicity; Ethnic minorities ^a^ |
| Household Registration | Rural; Urban |
| Marital Status | Married and living together; Single ^b^ |
| Region | Sichuan Basin; Yungui Plateau; Qinghai-Tibet Plateau |
| Education Level | Elementary school and below; Junior high school and high school; Associate degree or higher |
| Annual household income (¥) | <20,000; 20,000-100,000; >100,000 |

Note: “a” refers to the Tibetan, Yi, Miao, Bai, Dong, and Buyi ethnic groups. “b” refers to marital status including widowed, divorced, separated, and never married.

Table S4: Basic characteristics of study population grouped by CVH level

| Characteristics | Low CVH | Moderate CVH | High CVH | P-value |
| --- | --- | --- | --- | --- |
|  | N=11138 | N=62823 | N=11341 |  |
| Age group, N(%) |  |  |  | <0.001 |
| <50 | 2727(24.48) | 27799(44.25) | 8896(78.44) |  |
| ≥50 | 8411(75.52) | 35024(55.75) | 2445(21.56) |  |
| Gender, N(%) |  |  |  | <0.001 |
| Male | 7604(68.27) | 24949(39.71) | 1483(13.08) |  |
| Female | 3534(31.73) | 37874(60.29) | 9858(86.92) |  |
| Ethnicity, N(%) |  |  |  | <0.001 |
| Han ethnicity | 6382(57.30) | 36677(58.38) | 7899(69.65) |  |
| Ethnic minorities ^a^ | 4756(42.70) | 26146(41.62) | 3442(30.35) |  |
| Household Registration, N(%) |  |  |  | <0.001 |
| Rural | 7071(63.49) | 41863(66.64) | 6982(61.56) |  |
| Urban | 4067(36.51) | 20960(33.36) | 4359(38.44) |  |
| Marital Status, N(%) |  |  |  | <0.001 |
| Married and living together | 9489(85.19) | 55789(88.80) | 10404(91.74) |  |
| Single ^b^ | 1649(14.81) | 7034(11.20) | 937(8.26) |  |
| Region, N(%) |  |  |  | <0.001 |
| Sichuan Basin | 5667(50.88) | 29499(46.96) | 5880(51.85) |  |
| Yungui Plateau | 4598(41.28) | 28531(45.41) | 5037(44.41) |  |
| Qinghai-Tibet Plateau | 873(7.84) | 4793(7.63) | 424(3.74) |  |
| Education Level, N(%) |  |  |  | <0.001 |
| Elementary school and below | 4470(40.13) | 23026(36.65) | 3045(26.85) |  |
| Junior high school and high school | 5389(48.38) | 31768(50.57) | 6163(54.34) |  |
| Associate degree or higher | 1279(11.48) | 8029(12.78) | 2133(18.81) |  |
| Annual household income (¥), N(%) |  |  |  | <0.001 |
| <20,000 | 6393(57.40) | 33009(52.54) | 4000(35.27) |  |
| 20,000-100,000 | 3945(35.42) | 23321(37.12) | 5052(44.55) |  |
| >100,000 | 800(7.18) | 6493(10.34) | 2289(20.18) |  |
| Status of GSD, N(%) |  |  |  | <0.001 |
| No | 10066(90.38) | 58212(92.66) | 10827(95.47) |  |
| Yes | 1072(9.62) | 4611(7.34) | 514(4.53) |  |
| Age (years), mean ± SD | 57.56±10.91 | 52.00±11.25 | 43.25±8.90 | <0.001 |
| Monocyte count(10⁹/L), mean ± SD | 0.40±0.17 | 0.34±0.14 | 0.30±0.12 | <0.001 |
| Lymphocyte count(10⁹/L), mean ± SD | 1.99±1.12 | 1.82±0.61 | 1.67±0.50 | <0.001 |
| Neutrophil count(10⁹/L), mean ± SD | 4.15±1.78 | 3.75±1.36 | 3.57±1.31 | <0.001 |
| SIRI Index, mean ± SD | 0.93±0.70 | 0.79±0.61 | 0.71±0.58 | <0.001 |
| Ln(GGT)，mean ± SD | 3.77±0.78 | 3.27±0.71 | 2.82±0.55 | <0.001 |

Note: “a” refers to the Tibetan, Yi, Miao, Bai, Dong, and Buyi ethnic groups. “b” refers to marital status including widowed, divorced, separated, and never married. Abbreviation: CVH, cardiovascular health; GSD, gallstone disease; SIRI, systemic inflammatory response index; GGT, gamma-glutamyl transferase.

Table S5: Correlation analysis between LE8 series indicator score and GSD

| LE8 Series Indicator Score | Model1 | | Model 2 | | Model 3 | |
| --- | --- | --- | --- | --- | --- | --- |
|  | OR(95%CI) | P-value | OR(95%CI) | P-value | OR(95%CI) | P-value |
| Healthy behaviors |  |  |  |  |  |  |
| Sleep Score | 0.999(0.998,1000) | 0.042 | 1.001(1.000,1.001) | 0.300 | 1.001(1.000,1.002) | 0.250 |
| Diet Score | 1.000(0.999,1.000) | 0.418 | 1.000(0.999,1.001) | 0.931 | 1.000(0.999,1.001) | 0.754 |
| PA Score | 0.991(0.990,0.992) | <0.001 | 0.992(0.991,0.993) | <0.001 | 0.994(0.993,0.995) | <0.001 |
| Nicotine Exposure Score | 1.002(1.001,1.002) | <0.001 | 1.000(1.000,1.001) | 0.292 | 1.000(0.999,1.001) | 0.719 |
| Health Factors |  |  |  |  |  |  |
| BMI Score | 0.988(0.987,0.989) | <0.001 | 0.988(0.987,0.989) | <0.001 | 0.988(0.987,0.989) | <0.001 |
| Blood Pressure Score | 0.995(0.994,0.996) | <0.001 | 0.996(0.995,0.997) | <0.001 | 0.996(0.995,0.997) | <0.001 |
| Blood Glucose Score | 0.994(0.993,0.995) | <0.001 | 0.996(0.995,0.997) | <0.001 | 0.996(0.995,0.997) | <0.001 |
| Blood Lipid Score | 0.996(0.995,0.997) | <0.001 | 0.997(0.996,0.998) | <0.001 | 0.997(0.996,0.998) | <0.001 |

Model1: No covariates adjusted; Model 2: Adjusted for age and gender; Model 3: Adjusted for age, gender, ethnicity, household registration, marital status, region, education level, and annual household income. Abbreviation: LE8, life’s essential 8; GSD, gallstone disease; PA, Physical activity; BMI, body mass index; OR, odds ratio; CI, confidence interval.

Table S6: Sensitivity analysis of the relationship between LE8 and GSD

| LE8 Score | Exclude participants with a history of CVD | |  | Continue to exclude participants with cholestasis | |
| --- | --- | --- | --- | --- | --- |
|  | OR(95%CI)* | P-value |  | OR(95%CI)* | P-value |
| For each 10-point increase in LE8 | 0.834(0.815,0.853) | P<0.001 |  | 0.837(0.816,0.858) | P<0.001 |
| Classified |  |  |  |  |  |
| Low CVH | Ref. |  |  | Ref. |  |
| Moderate CVH | 0.742(0.688,0.800) | P<0.001 |  | 0.741(0.682,0.804) | P<0.001 |
| High CVH | 0.467(0.414,0.526) | P<0.001 |  | 0.479(0.421,0.545) | P<0.001 |

Note: “*” indicates adjustment for age, gender, ethnicity, household registration, marital status, region, education level, and annual household income. Abbreviation: LE8, life’s essential 8; GSD, gallstone disease; CVD, Cardiovascular disease; OR, odds ratio; CI, confidence interval.

Table S7: Sensitivity analysis of the mediating effect of SIRI on the relationship between LE8 and GSD

|  | independent variable | Mediating variable | Total effect | Direct effect | Indirect effects | Proportion of Mediation |
| --- | --- | --- | --- | --- | --- | --- |
|  |  |  | Coefficient  (95%CI) | Coefficient  (95%CI) | Coefficient  (95%CI) |  |
| Exclude participants with a history of CVD | For each 10-point increase in LE8 | SIRI | -0.0259  (-0.0311,-0.0212) | -0.0252  (-0.0304,-0.0205) | -0.0007  (-0.0010,-0.0004) | 2.69% |
|  |  |  |  |  |  |  |
| Continue to exclude participants with cholestasis | For each 10-point increase in LE8 | SIRI | -0.0207  (-0.0258,-0.0165) | -0.0201  (-0.0251,-0.0159) | -0.0006  (-0.0009,-0.0004) | 2.97% |

Abbreviation: LE8, life’s essential 8; SIRI, systemic inflammatory response index; GSD, gallstone disease; CVD, Cardiovascular disease; CI, confidence interval.

Table S8: Sensitivity analysis of the mediating effect of GGT on the relationship between LE8 and GSD

|  | independent variable | Mediating variable | Total effect | Direct effect | Indirect effects | Proportion of Mediation |
| --- | --- | --- | --- | --- | --- | --- |
|  |  |  | Coefficient  (95%CI) | Coefficient  (95%CI) | Coefficient  (95%CI) |  |
| Exclude participants with a history of CVD | For each 10-point increase in LE8 | GGT | -0.0257  (-0.0309,-0.0210) | -0.0242  (-0.0294,-0.0196) | -0.0015  (-0.0019,-0.0010) | 5.57% |
|  |  |  |  |  |  |  |
| Continue to exclude participants with cholestasis | For each 10-point increase in LE8 | GGT | -0.0205  (-0.0256,-0.0163) | -0.0192  (-0.0241,-0.0150) | -0.0013  (-0.0018,-0.0009) | 6.40% |

Abbreviation: LE8, life’s essential 8; GGT, gamma-glutamyl transferase；GSD, gallstone disease; CVD, Cardiovascular disease; CI, confidence interval.


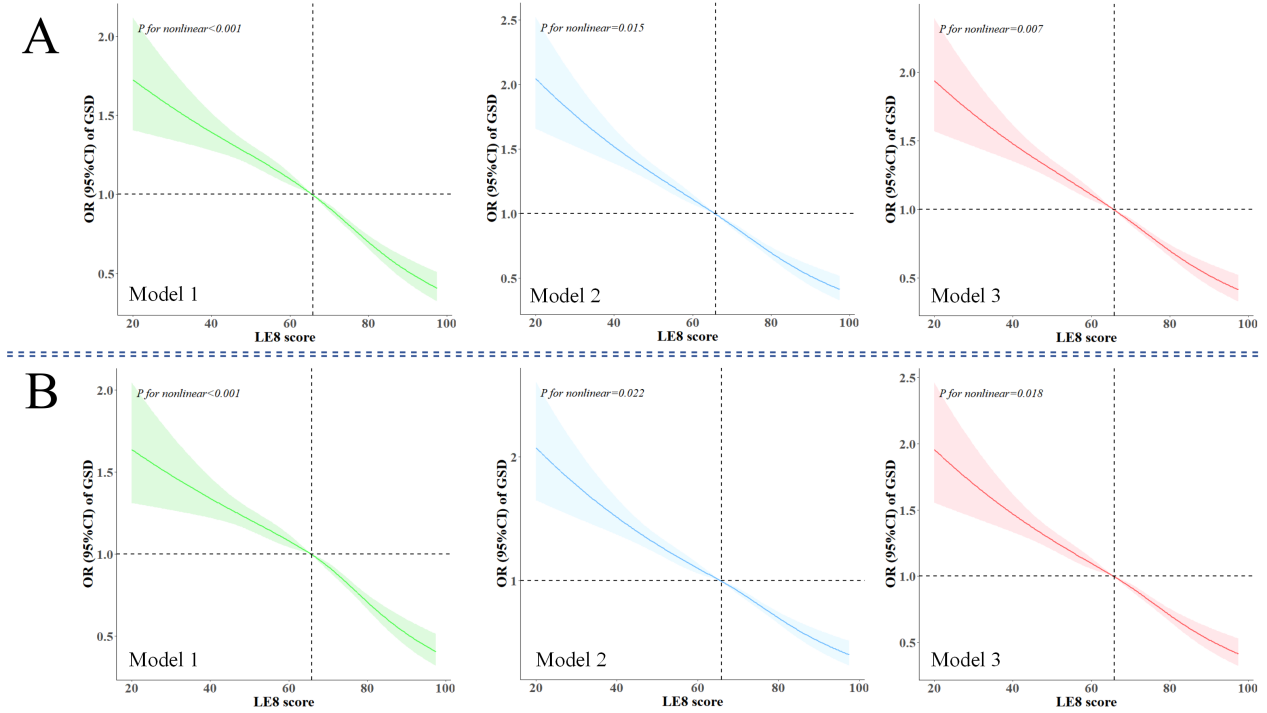


Figure S1: Sensitivity analysis of the dose-response relationship between LE8 and GSD risk. Note: “A” excludes participants with a history of CVD; “B” Continue to excludes participants with cholestasis.The solid line represents the fitted smooth curve between variables, and the shaded area indicates the fitted 95% confidence interval. Model1: No covariates adjusted; Model 2: Adjusted for age and gender; Model 3: Adjusted for age, gender, ethnicity, household registration, marital status, region, education level, and annual household income. Abbreviation: LE8, life’s essential 8; GSD, gallstone disease; OR, odds ratio; CI, confidence interval; CVD, Cardiovascular disease.


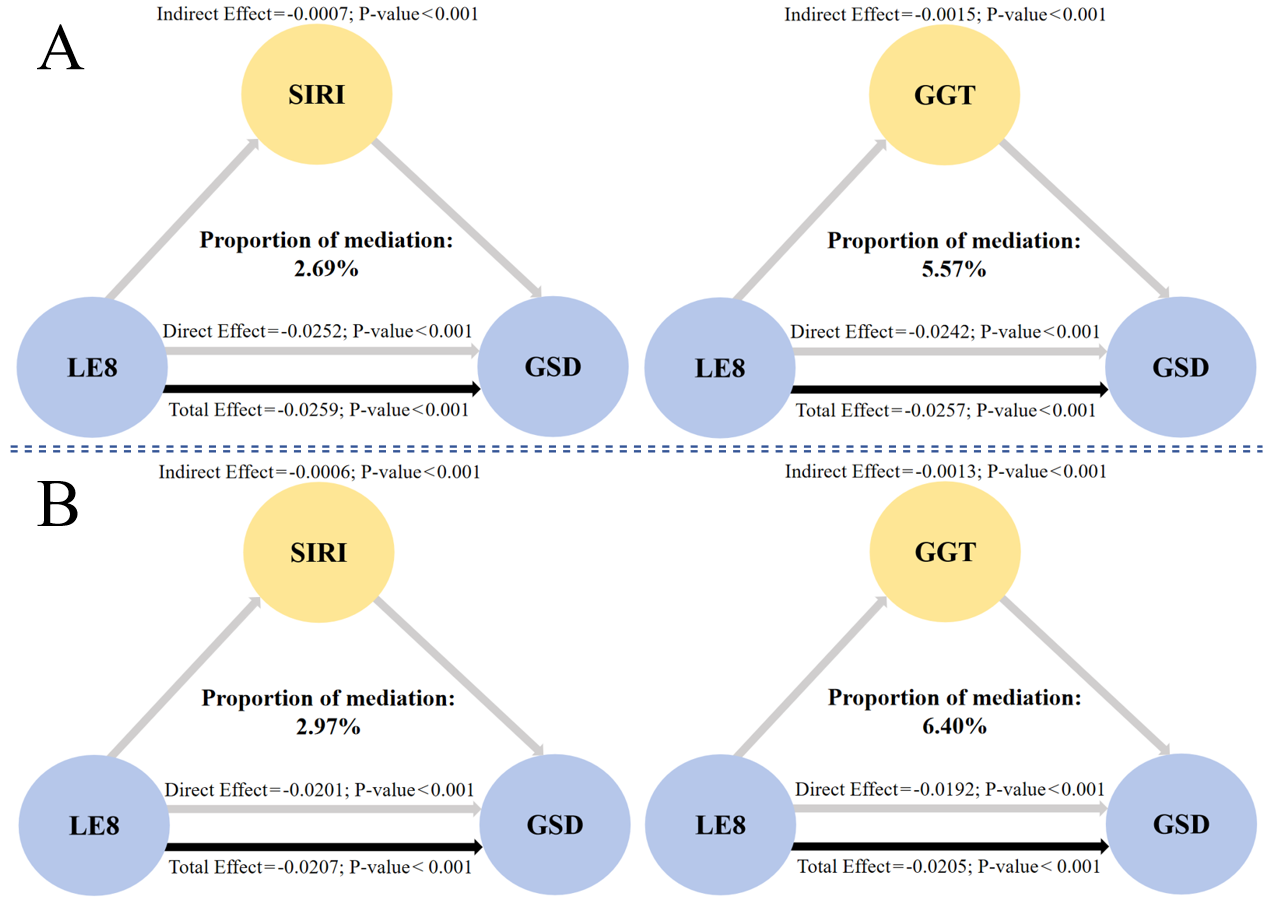


Figure S2: Sensitivity analysis of the mediating effect pathways of inflammation and oxidative stress between LE8 and GSD. Note: “A” excludes participants with a history of CVD; “B” Continue to excludes participants with cholestasis. For each 10-point increase in LE8 as the independent variable, GSD serves as the dependent variable, with SIRI and GGT acting as mediating variables.The total effect represents the overall relationship between LE8 and GSD that is unaffected by mediating variables. The direct effect indicates the direct relationship between LE8 and GSD after controlling for SIRI or GGT. The indirect effect reflects the influence of LE8 on GSD mediated through SIRI or GGT. Abbreviation: LE8, life’s essential 8; SIRI, systemic inflammatory response index; GGT, gamma-glutamyl transferase; GSD, gallstone disease.
